# Variations of Ethmoid Roof in the Iranian Population- A Cross Sectional Study

**DOI:** 10.22038/ijorl.2019.37340.2220

**Published:** 2020-05

**Authors:** Maryam Moradi, Bahare Dalili

**Affiliations:** 1 *Department of Radiology, School of Medicine, Isfahan University of Medical Sciences, Isfahan, Iran.*; 2 *School of Medicine, Isfahan University of Medical Sciences, Isfahan, Iran.*

**Keywords:** Anatomy, Classification, Ethmoid sinus

## Abstract

**Introduction:**

This study aimed to investigate the distribution of ethmoid roof variation and symmetry according to Keros classification.

**Materials and Methods:**

This cross-sectional study assessed the paranasal CT scans of 600 patients over 18 years of age with no history of surgery, trauma, or localized fracture in the ethmoid, nose, and anterior skull base. The lateral lamella of the cribriform plate (LLCP) sizes were measured and classified as Keros type I (1-3 mm), II (4-7mm), and III (8-16mm). Moreover, the symmetry was surveyed in accordance with the LLCP measurements on both sides of the ethmoid roof. These variations were analyzed regarding gender and age.

**Results:**

In total, 600 patients participated in this study out of whom 311 cases were male. According to the results, the mean age of the participants was 37.50±16.63 years. Furthermore, the mean values of the LLCP height for the right and left sides were 4.17±1.69 and 4.93±1.97 mm, respectively. Moreover, the asymmetry was observed in 38.3% of the cases, and they were classified as 36.7% Keros type I, 50.5% Keros type II, and 12.8% Keros type III.

**Conclusion:**

Keros type II and symmetry were the most common variations in this study. In addition, these variations were independent of age and gender.

## Introduction

Computed Tomography (CT) scan is the standard method for sinonasal imaging, and radiological examinations are not only for diagnostic purposes of sinonasal pathologies but also the description of their anatomy, which increase the vision of surgeons for several interventions in this area, such as endoscopic sinus surgery (ESS) (1). The ESS is a safe surgical procedure that is commonly used for nasal polyps, rhinosinusitis (acute or chronic), neoplasms, and developmental abnormalities (2). This procedure is operated with an endoscope, which is directed in the fovea ethmoidalis. The fovea ethmoidalis, a part of the frontal bone orbital plate, forms the roof of the ethmoidal labyrinth that separates ethmoidal cells from the anterior cranial fossa. Lateral lamella of the cribriform plate (LLCP) is attached to the medial side of fovea ethmoidalis (3). 

The LLCP is the thinnest and most vulnerable structure in the skull base that can be perforated and fractured easily through ESS and leads to further complications (3,4). The knowledge of anatomical variations helps surgeons to operate more accurately with fewer complications (5,6). 

Keros, a Croatian physician, introduced a method in 1962 to categorize the depth of olfactory fossa defining variations in the horizontal level of the cribriform plate of the ethmoid bone (7). According to the length of LLCP, three types have been described in this regard. Keros Type I with 1-3 mm depth of olfactory fossa defines a short lateral lamella with the ethmoid roof and cribriform plate almost in the same plane. Olfactory fossa with a depth of 4-7 mm is classified as Keros type II with longer lateral lamella. Moreover, Keros type III represents an olfactory fossa with a depth of 8-16 mm, and the ethmoid roof is significantly higher than the cribriform plate. Keros type III is also known as “dangerous ethmoid” due to the higher incidence of complications in this type (2).

Asymmetry is another common variation that is important to be investigated before ESS surgery showing that exact similar procedure should not be applied in both sides (8). Preoperative assessment of these parameters gives the surgeons a better perception of the roadmap before surgery. Distribution of these variations is different depending on gender and ethnicity (9). Due to the dearth of research in this regard in Iran and lack of studies on asymmetry variation and its distribution, this study was conducted to investigate the prevalence of Keros types based on gender and age, as well as asymmetry distribution in an Iranian population.

## Materials and Methods

This cross-sectional study was conducted at Al-Zahra University Hospital, Isfahan, Iran, between February and September 2018. The study protocol was approved by the Ethics Committee of Isfahan University of Medical Sciences, Isfahan, Iran. Informed consent was not required since this retrospective analysis was performed on clinically indicated CT scan studies; however, when the image assessments started, the confidentiality of the data was taken into consideration in the ward. The patients were selected by a simple random sampling method. This study included patients who were over 18 years of age with no history of surgery, trauma, or localized fracture in the ethmoid, nose, and anterior base skull base. On the other hand, the patients whose CT scans had poor resolution or quality and those who had sinonasal tumors, nasal polyps, serious rhinosinusitis, or any sinus disorder complicating the measurements were excluded from the study. All CT scan images were obtained with a single multi-detector 64-slice CT scanner (VCT 64 Slice, GE, United States). The CT scan technical features were as follows: Kv=120, Auto mA (50-300), Rotation time=0.5 sec, Filter: Bone, Slice thickness: 0.625 mm with 50% overlap, Reconstructions: 3-5 mm, Pitch=0.5, Field of view (FOV)=240-50 mm, Window level= 500, Window width: 2500, Filter: bone plus. 

The images were reconstructed using computerized software in Picture Archiving and Communication System. Moreover, the measurements were performed by one adjacent person in this system using the distance measuring tool. In order to measure the size of LLCP, a baseline was drawn between two infraorbital foramina on the floor of both orbital cavities. Subsequently, two parallel perpendicular lines were drawn from the medial ethmoid roof point equivalent to the medial part of the ethmoid roof and another one from the lowest part of the cribriform plate. These two lines were measured, and the result of their subtraction was considered the LLCP size. These measurements were also performed for another side using crista Galli as the line of symmetry. The LLCP sizes in ranges of 1-2.99, 3-6.99, and 7-16 were categorized as Types I, II, and III of Keros classification, respectively. Figure 1 illustrates the LLCP.

The asymmetry was confirmed when the subtraction of the LLCP sizes of the left and right sides was more than1mmwith the flattening of the fovea on one side (10). Demographic characteristics, such as gender and age were obtained from the patients' files. The sample size was calculated 600 with a confidence interval of 95%, accuracy of 3.5%, and photovoltaic =76% according to a study carried out by Kaplanglu et al. (11)

The data were analyzed in SPSS software (version 25.0) (SPSS Inc., Chicago, IL, USA). Moreover, the prevalence of Keros types and asymmetry were reported with the percentages, and the Keros types were analyzed according to age and gender using the Chi-square test. A p-value less than 0.05 was considered statistically significant.

## Results

In total, 600 patients were included in this study out of which 311 cases were male. According to the results, the mean age of the patients was 37.50±16.63 years, and the mean values of the LLCP height for the right and left sides were 4.17±1.69 and 4.93±1.97 mm, respectively. Moreover, asymmetry was observed in 38.3% of the cases. There was no significant difference between males and females regarding age, the height of the right and left sides, and asymmetry (P>0.05) (Table.1).

**Table 1:** Age, lateral lamella of the cribriform plate height, and asymmetry based on gender

**Variables **		**Gender**		**Overall**	**P-value**
		**Male (n=311)**	**Female (n=289)**		
Age		37.36±16.50	37.65±16.80	37.50±16.63	0.68
Height	Right	4.25±1.75	4.08±1.62	4.17±1.69	0.07
	Left	4.98±2.04	4.87±1.89	4.93±1.97	0.08
Asymmetry		126(40.5%)	104(36%)	203(38.3%)	0.25

The cases were classified as 36.7% Keros type I, 50.5% Keros type II, and 12.8% Keros type III. The results revealed no significant difference between males and females in terms of Keros types (P=0.17) (Table.2).

**Table 2:** Keros types of right and left sides based on gender

**Variables **		**Gender**		**Overall**	**P-value**
		**Male (n=311)**	**Female (n=289)**		
Keros types	I	230(37%)	210(36.3%)	440 (36.7%)	0.17
	II	298(47.9%)	308(53.3%)	606(50.5%)	
	III	94(15.1%)	60(10.4%)	154(12.8%)	

The mean ages of the patients with asymmetry and symmetry were 39.08±17.40 and 34.97±15.01 years, respectively. Moreover, the LLCP height for the right and left sides were 3.43±0.93 and 6.51±1.87mm in the patients with asymmetry, and the corresponding values were obtained at 4.64±1.88 and 3.95±1.28 mm in the patients with symmetry. It is worth mentioning that the Keros types had no significant correlations with age and gender (P>0.05).

## Discussion

This study aimed to investigate the distribution of different variations of the ethmoid roof in the Iranian population. Key variations that were targeted to describe differences in the ethmoid roof included LLCP, size, and symmetry. The LLCP size was defined and reported according to Keros classification. The majority of the patients were classified as Keros type II, and symmetry was also more prevalent than asymmetric ethmoid roofs on both sides. Several studies were conducted to explore Keros types in different populations. Kaplangolu et al. reported 13.4% Keros type I, 76.1% Keros type II, and 10.5% Keros type III in the Turkish population (11). In a study conducted by Souza et al. on 200 patients, the cases were classified as 26.5% Keros type I, 73.33% Keros type II, and the remaining minority 0.5% Keros type III (12). In the same line, a study on the Filipino population showed that the majority of the patients were classified as 81.6% Keros type I, 17.9% Keros type II, and 0.5% Keros type III (1). These studies were conducted on different ethnicities and demonstrated that the distribution of Keros classes was related to the ethnicity of the patients. Gender is another factor that can have effects on the results of the study, which is almost equal in different studies. Additionally, in the present study, there was no significant difference between males and females regarding LLCP heights, asymmetry, and Keros types.

According to the results of this study, the majority of our patients were categorized as Keros type II (50.5%) followed by Keros type I (36.7%) and Keros type III (12.8%), which was the least prevalent. These results were in line with the findings of most studies; however, Alazzawi et al. reported 80% and 20% of the prevalence rates for type I and II, respectively (13). Alazzawi et al. conducted a study on three different ethnicities, namely Malay, Chinese, and Indian. They reported Keros type I (80%) as the most prevalent followed by no case of Keros type III. They also found a significant difference between males and females in terms of Keros types II introducing males as majorities in this type. According to a study performed by Paber et al., Keros type I was the most common type in the Filipino population (1).

The present study showed that the majority of patients had Keros type II expressing a moderate risk of complications in the ESS surgery. Moreover, there were no significant differences between the age groups in terms of the distribution of Keros types. Asymmetry was observed in 38.3% of the patients, which was consistent with the findings of other studies. In a study performed by Lebowitz et al. asymmetry in the ethmoid bone was observed in 9.5% of the patients (10). Moreover, Adeel et al. stated that they found asymmetry in 10% of their patients (8). On the other hand, Kaplangolu et al. reported asymmetry in 80% of their cases (11). The sample size in the studies carried out by Adeel et al. and Lebowits et al. was lower than that in this study and the study performed by Kaplangolu. Moreover, there were differences in target ethnicities and the design of the study. In a study conducted by Babuer al., the frequency of Keros type I, II, and III were 17.5%, 74.6%, and 7.9%, respectively. Moreover, the mean value of the olfactory fossa was estimated at 5.26, and type III was more prevalent in males and right side followed by 75% asymmetry in olfactory fossa (14). 

## Conclusion

According to the results of this study, Keros type II was the most common type in Keros classification in the Iranian population, and symmetry was more prevalent in these people. Therefore, it is suggested that further studies be performed with a multicentric design on the Iranian population due to the existence of different ethnicities in this country. The present study included adult patients who were over 18 years of age. Therefore, it is necessary to conduct studies on adolescents in the Iranian population to clarify the distribution of Keros types in this age group.
